# Impulsivity and aggression in suicide ideators and suicide attempters of high and low lethality

**DOI:** 10.1186/s12888-022-04398-w

**Published:** 2022-12-01

**Authors:** Silje Støle Brokke, Nils Inge Landrø, Vegard Øksendal Haaland

**Affiliations:** 1 Department of Psychiatry, Sørlandet Hospital, Po box 416, N-4604 Kristiansand, Norway; 2 Clinical Neuroscience Research Group, Department of Psychology, Faculty of Social Sciences, University of Oslo, Oslo, Norway; 3 Division of Mental Health, Sørlandet Hospital, Kristiansand, Norway

**Keywords:** Aggression, Impulsivity, Suicidal behaviour, Suicide attempt, Suicide ideation

## Abstract

**Background:**

Impulsivity and aggression have been associated with all forms of suicidal behaviour and linked to theories of suicide capability. There is a need to clarify the role of impulsivity and aggression in the progression from suicidal thoughts to suicide attempts and suicide.

**Method:**

In this naturalistic cross-sectional study, suicide ideators (35), low lethal suicide attempters (37), and high lethal suicide attempters (26) were compared with the Columbia-suicide severity rating scale (C-SSRS), Barratt impulsiveness scale (BIS-11), and the Buss & Perry aggression questionnaire (AQ).

**Results:**

Physical aggression score (*p* = 0.032) contributed to the difference between predicted low lethal suicide attempt and predicted high lethal suicide attempt. This model predicting physical aggression showed a fairly weak positive relationship (OR = 1.1) to high lethal attempt and explained 13% of the variance so there is a need for further replications to verify these results. Impulsive behaviour scores in females were significantly higher in the low lethal suicide attempt group compared to suicide ideators (F(2.51) = 3.47, *p* = 0.039, η²= 0.12). Hostility aggression in females was significantly higher in the high lethal suicide attempters compared to suicide ideators (F(2.52) = 3.53, *p* = 0.037, η² = 0.12). Physical aggression scores in females were significantly higher in the high lethal attempters compared to suicide ideators (F(2.52) = 6.79, *p* = 0.002, η²= 0.21). When these analyses were conducted without the participants who died in suicide, men in the high lethal attempt group scored significantly higher than men in the low lethal attempt group (F(2.37) = 3.8, *p* = 0.031, η² = 0.17), but men did not differ in aggression and impulsivity scores in other comparisons.

**Conclusion:**

Suicide prevention should address physical aggression, as high levels can be associated with high lethal attempts. Assessment of suicidal patients should address impulsive behaviour with the insight that it can be more prominent in female low lethal suicide attempters. It could be that assessment and treatment of suicidal patients should be tailored differently for men and women. Aggression as a feature of suicide capability could be the link that makes suicide possible.

## Background

The progression from suicidal thoughts to suicide attempt is now understood as a distinct process [1]. Since most traditional risk factors for suicide are risk factors for both suicide ideation and suicide attempt [2], it is important to understand what differentiates suicide attempters from suicide ideators [3]. Many people have suicidal thoughts, but most people will never attempt suicide [4]. It is essential to understand why some people do [5].

Research suggest that most people who die by suicide suffered from one or more mental illness at time of death [6, 7]. A systematic review and meta-analysis of suicidal behaviour found the most prevalent mental illness of fatal and non-fatal suicide attempt to be mood disorders [8]. A systematic review and meta-analysis of people with serious mental illness found suicide rates of people with major depressive disorder, bipolar disorder and schizophrenia to be high [9].

Impulsivity and aggression are two personality traits that have been linked to all forms of suicidal behaviour. Impulsivity includes a broad range of behaviours like self-regulation, planning, responding before considering consequences, sensation seeking, risk taking, low inhibition, and preference for immediate rewards [10]. The concept of aggression can include behaviours such as verbal aggression, physical aggression, hostility, and anger [11]. There are multiple epidemiological, clinical, retrospective, prospective, and family studies that have found a strong link between aggression and completed suicide [10]. A meta-analysis showed that aggression and impulsivity are highly correlated with suicidal behaviour across different psychiatric and non-psychiatric samples [12].

In the study of the suicide process, it is now evident there exist gender differences in suicidal behaviour. A main gender difference, often referred to as the ‘gender paradox’, is that women have more suicide attempts than men do, but men more often die by suicide [13]. Men have been found to engage in more serious suicide attempts than women [14] and men more often use high lethal methods [15]. Research also indicates that there is a difference between men and women on personality characteristics like impulsivity and aggression. Men tend to be more impulsive than women [16] and men also tend to engage in more aggressive behaviours than women and respond more often with physical aggression to stress, frustration, and negative emotions [17].

The ideation-to-action framework can help in understanding the impact of impulsivity and aggression on suicidal behaviour. The ideation-to- action framework includes theories that argue that the development of suicide ideation, and progression from suicide desire to suicide attempt are distinct processes with distinct explanations [18]. One of these theories, called the three-step theory (3ST) includes 3 steps to describe the progression from ideation to action [18]. The first step includes suicide ideation which can develop from psychological pain and hopelessness. The second step highlights the importance of connectedness, when the degree of pain and hopelessness can affect the ability to connect in relationships, to activities, and to meaning in life. Step three is when the progression from ideation to attempt can be facilitated by dispositional, acquired, and practical contributors that can build a capacity to attempt suicide. We are biologically and evolutionary wired to avoid pain, injury, and death, so to build a capability to go against our natural instinct to survive is necessary for a suicide attempt to be possible. How pain sensitive a person is could be a predisposition, while trauma, drugs, and self-harm could build pain tolerance, and the place where one works could play a role in the access to lethal means [19].

An increasing body of research supports suicide capability as a construct that differentiates suicide attempters from suicide ideators [1]. Trait impulsivity has been described as acting as a distal risk factor via capability, as individuals with high levels of trait impulsivity can experience more injuries and engage more in suicidal behaviour, and by doing so experience more habituation to pain [20]. Outward-directed aggression can also be linked to suicide capability as outward-directed aggression has been reported as higher in suicide attempters compared to suicide ideators [21]. In a study of characteristics of people who died by suicide in prison, it was found that close to 18% had an episode of agitated behaviour within the week before suicide [22].

Another study found that impulsivity differentiated suicide attempters from non-attempters in major depressive disorder and also found impulsive and aggressive traits to be strongly correlated in suicide attempters but not in non-suicide attempters [23]. A study of impulsivity and aggression in college students found a difference in cognitive impulsivity and self-oriented attack when comparing suicide ideators with suicide attempters, and that suicide attempters scored higher than suicide ideators [24].

To understand the process from suicidal thoughts to suicide death, it can be especially valuable to also include individuals who have died in suicide and individuals who have survived a high lethal suicide attempt. Research indicates that the lethality of previous suicide attempts predicts lethality of future attempts [25]. A study comparing suicide ideators, suicide attempters of low medical seriousness, and suicide attempters of high medical seriousness found that higher levels of aggression, impulsivity, and mental pain differentiated attempters from suicide ideators. They found communication difficulties to be the only factor that differentiated medically serious attempters from medically non-serious attempters [26]. A systematic review of suicide attempters with different degree of seriousness concluded that the risk factors of interpersonal problems, impulsivity, and aggression can differentiate serious suicide attempts from less serious attempts [27]. However, there are also studies that have found no difference between high lethal suicide attempters and low lethal suicide attempters on the measures of impulsivity and aggression [28].

A main aim of the current study is to seek an understanding of the relation between impulsivity, aggression, and suicidal behaviour. The study will also add knowledge to the scarce literature on lethality nuances in suicide attempts, and gender differences in suicidal behaviour. Suicide ideators, low lethal suicide attempters, and high lethal suicide attempters will be compared on suicide-related characteristics, clinical characteristics, and measures of aggression and impulsivity. It is predicted that suicide attempters will score higher than suicide ideators on impulsivity and aggression, and it is predicted that men will score higher than women on most measures. It is uncertain whether there will be a difference between low lethal suicide attempters and high lethal suicide attempters so analysis will explore these associations. As high lethal attempters is the most vulnerable group for suicide, the main analysis will focus on associations related to this group.

## Method

### Participants

The participants were acute psychiatric patients who were referred to the crisis resolution team at Sørlandet hospital in southern Norway. The crisis resolution team aims to help people who are experiencing mental health crisis, usually related to suicidality and acute mental illness. The study was designed as a natural cross-sectional study of suicidality in acute psychiatric patients. The inclusion period was from May 2014 to August 2017. Most of the patients were outpatients, but some were inpatients. There were 367 patients who were invited to participate, of which 227 agreed and, in the end, 98 patients completed all measurements included for the analyses. When comparing the patients who agreed to participate to the patients that did not want to participate, there were no significant differences in gender, age, or depression. There were also no significant differences in age, gender, or depression for the ones who completed all measurements and the ones who only completed some of the measurements. The participants included in this specific paper were patients with suicide ideation, low lethal suicide attempt, and high lethal suicide attempt who had completed a set of online questionnaires and a clinical interview regarding suicidal behaviour.

The suicide ideator group, the low lethal suicide attempt group and the high-lethal suicide attempt group were all identified through the Columbia-suicide severity rating scale (C-SSRS) [29]. The clinicians who conducted the interviews were all trained by a video training from developers of the C-SSRS and had completed at least one interview together with the main researcher of the project before they started the interview-rating independently. The patients that died by suicide after inclusion in the current study were identified through the Norwegian Cause of Death Registry combined with information from the patients’ medical records.

*The suicide ideator group* includes the participants who reported lifetime suicide ideation and no suicide attempt in C-SSRS, question 1–6 and assessment of actual attempt [29].

*The low lethal suicide attempt group* includes participants who reported suicide attempt, but where the suicide method was non-lethal. This information was obtained from C-SSRS, actual lethality, a score of either 0, ‘attempt will most likely not lead to harm’ or 1, ‘behavior will most likely lead to harm, but not death’[29].

*The high-lethal suicide attempt group* includes participants who described having survived a high lethal attempt (*n* = 21) as identified in C-SSRS, actual lethality, a score of 2, ‘attempt will most likely lead to death, also when medical assistance is available’[29] and participants who died in suicide (*n* = 5) during the project period. The decedents were one suicide ideator, one high lethal suicide attempter and three low lethal suicide attempters prior to their deaths.

The patients signed a consent form before inclusion that allowed the researcher to review the patients’ medical records in the timeframe from project start until project ended in 2022. Serious suicide attempters have been found to be epidemiologically very much like the people who have died by suicide [27]. When comparing high lethal suicide attempters with the patients that have died in suicide attempt, there were no significant differences between the clinical measures of the two groups.

### Measurement

The questionnaire and interview were all Norwegian versions.

### Barratt impulsiveness scale (BIS-11)

The Barratt impulsiveness scale (BIS-11) is the most frequently used self-report measure of impulsivity with good reliability and validity [30]. A Norwegian version of the scale was used in this study [31]. The questionnaire includes 30 questions on impulsiveness control and pathological impulsiveness. For each question, the response is given on a scale from 1 to 4 where 1 is ‘rare/never’, 2 is ‘now and then’, 3 is ‘often’, and 4 is ‘most of the time/always’. A study of the psychometric properties of a swedish version of BIS (the Swedish language is very similar to Norwegian) found good to excellent psychometric properties in a population of healthy controls, patients with substance abuse, and patients of ADHD [32]. Research has, however, suggested different factor structures for best fit. For this study we conducted a factor analysis that resulted in a three-factor model: cognitive impulsivity, behavioural impulsivity, and impatience/restlessness. This three-factor model has been supported in another recent factor analysis [33].

### Aggression questionnaire (AQ)

The aggression questionnaire (AQ) [11] is a self-report 28-item scale [11] that measures violent behavior. The questionnaire examines four different elements of violence: hostility (eight items), anger (seven items), verbal aggression (five items), and physical aggression (eight items). The responses are given on a Likert-type scale ranging from 1 to 5 where 1 is ‘extremely uncharacteristic of me’ and 5 is ‘extremely characteristic of me’. Higher scores indicate higher aggression. Reliability and validity have been found to be good [34]. A Swedish version [35] has been revised and has shown internal consistency between the Swedish and the American version [36].

### Montgomery-Åsberg depression rating scale (MADRS)

The MADRS [37] is a clinically assessed, standardized rating scale designed to assess symptoms of an ongoing depression. A Norwegian version of the rating scale has been translated by Prof. Ulrik Malt. The validity of the rating scale has been supported in several studies [38]. The rating scale includes 10 phenomena related to depression and clinicians assess each phenomenon using a rating scale ranging from 0 to 6.

### Columbia-suicide severity rating scale (C-SSRS)

The Columbia-suicide severity rating scale (C-SSRS) [29], Norwegian version 14.01.09. was selected to assess the severity of suicidal behaviour and, specifically to assess suicide ideation and suicide-attempt history. The interview is structured as a screening interview, with five questions on suicide ideation, seven questions on intensity of ideation, six questions on suicidal behavior, and two questions on lethality evaluations of actual attempts. The C-SSRS covers both suicidal behaviour during the previous month and lifetime history of suicidal behaviour for all the different questions. Suicide attempts are further categorized by the first, latest, and most deadly attempt. The lethality of the attempts is rated on a scale from ‘attempt will most likely not lead to harm’ (0), ‘behavior will most likely lead to harm, but not death’ (1) and ‘attempt will most likely lead to death, also when medical assistance is available’ (2). Most questions in the interview are *yes- or no*-based, some questions are age-based, and some questions ask for the number of occurrences of suicidal behaviour or age for onset of behavior. The interview has shown good convergent and divergent validity with other multi-informant suicidal ideation and behaviour scales, and it has been validated as a suitable assessment of suicidal ideation and suicidal behaviour in both clinical and research settings [39].

### Diagnoses

Diagnoses from ICD-10 classification of mental and behavioural disorders were reported from assessment when the participants first met the crisis resolution team. Some of the patients had already been diagnosed with a mental illness before referral and some of the patients did not meet the criteria of the ICD-10 classifications.

### Procedure

The participants were asked to participate in the study after a first meeting with clinicians of the crisis resolution team. All participants were given a written information form and the nature of the study was explained to them. The participants that agreed to participate were given a consent form to sign and informed that they had the right to withdraw from the study at any time.

The clinicians involved in the data collection were health professionals on the crisis resolution team, trained to complete the screening interview. The training included a video made by the developers of the C-SSRS in addition to observing at least one interview.

### Statistical analyses

Statistical analyses were performed using SPSS (version 28.0) for windows. A descriptive analysis was conducted to compare clinical characteristics and suicidal behaviour in the suicide ideator group, low lethal attempt group, and high lethal attempt group. Gender was separated for male and female impulsivity and aggression scores because the pattern of suicide attempts and completed suicide is different in males and females [40]. T-tests and chi-square tests were used where appropriate, and means and standard deviations were reported. The analyses of demographic and clinical characteristics were conducted for descriptive purposes only. A parametric analysis of variance with Bonferroni post hoc comparisons was used to compare the means of the scores of impulsivity and aggression in the three groups. A logistic regression analysis was conducted to test the predictor variable from the parametric analysis, and no further alpha adjustment for multiple comparisons was made.

## Results

### Clinical characteristics

Table 1 shows different clinical and suicide risk-related variables for suicide ideators, low lethal suicide attempters, and high lethal suicide attempters. Gender, age, depression, and age of first suicide thought were well matched. The most common diagnostic groups were affective disorders F30-F9 and neurotic and stress related disorders F40-F49 in all three groups. High lethal suicide attempters had fewer years of education (*p* < 0.051) than suicide ideators. Ideators were less likely to self-harm than low lethal attempters (*p* < 0.036). Ideators had lower suicide risk than high lethal suicide attempters (*p* < 0.020).

**Table 1:** Characteristics of suicide ideators, low lethal suicide attempters and high lethal suicide attempters

	Suicide ideator	Low lethal suicide attempter	High lethal suicide attempter
**N(%)**	35(36)	37(38)	26(27)
**Female(%)**	19(54)	22(59)	13(50)
**Age(** ***SD)***	39(15)	34(15)	34(15)
**Depression score(SD)**	24.2(7.9)	25.2(7.3)	25.2(6.7)
**Years of education(SD)**	13.7(3.6)	13.1(2.5)	**11.7(3.7)**
**Age of first suicide thought (SD)**	25.6(16.0)	17.5(16.3)	21.9(14.6)
**Diagnosis F10-F19**	3(9%)	1(3%)	4(15%)
**Diagnosis F20-F29**	0(0%)	0(0%)	1(4%)
**Diagnosis F30-F39**	13(37%)	19(51%)	10(38%)
**Diagnosis F40-F49**	10(29%)	8(22%)	7(27%)
**Diagnosis F50**	1(3%)	0(0%)	0(0%)
**Diagnosis F60-F69**	3(9%)	7(20%)	2(8%)
**Diagnosis F80-F89**	2(6%)	0(0%)	0(0%)
**Diagnosis F90-F98**	1(3%)	1(3%)	0(0%)
**No diagnosis**	2(6%)	1(3%)	2(8%)
**Self-harm lifetime**	**0.3**	0.6	0.5
**Suicide risk level, 1–5**	**4.2**	4.6	4.7
**Total suicide attempts**	0	8.7	7.0

#### Impulsivity and aggression of male and female suicide ideators, low lethal attempters, and high lethal attempters

Table 2 shows different index scores of impulsivity and aggression for male (*n* = 45) and female (*n* = 55) suicide ideators, low lethal suicide attempters, and high lethal suicide attempters. Female patients in the high lethal suicide attempt group scored higher on impulsive behaviour (F(2.51) = 3.47, *p* = 0.039, η²= 0.12) than suicide ideators. No significant differences were found between the three groups on other measures of impulsivity for males and females. Female patients in the high lethal attempt group scored higher on physical aggression than suicide ideators (F(2.52) = 6.79, *p* = 0.002, η²= 0.21). Female patients in the high lethal suicide attempt group scored higher on hostility than the suicide ideator group (F(2.52) = 3.53, *p* = 0.037, η² = 0.12). No differences were found in other measures of aggression for male or female suicidal behaviour groups. A difference between men and women was found for the index score of verbal aggression (F(1.98) = 4.52, p = 0.035, η² = 0.19) and physical aggression (F(1.98) = 17.0, *p* = 0.010 η² = 0.36). Men scored higher than women did on the two aggression indexes. When these analyses were conducted without the participants who died in suicide, men in the high lethal attempt group scored significantly higher than men in the low lethal attempt group (F(2.37) = 3.8, *p* = 0.031, η² = 0.17).

**Table 2:** Impulsivity and aggression of male and female suicide ideators, low lethal attempters, and high lethal attempters

Measure	Construct	Suicide ideator	Low lethal suicide attempt	High lethal suicide attempt	F	*P*
**BIS-female**	**Cognitive impulsivity**	24.1(5.6)	24.5(6.1)	21.9(7.0)	0.75	0.478
**BIS-male**	**Cognitive impulsivity**	22.1(5.3)	23.0(4.8)	23.7(5.0)	0.35	0.707
**BIS-female**	**Impulsive behaviour**	**16.0(4.3)**	**20.5(6.6)**	**19.7(5.7)**	**3.47**	**0.039a**
**BIS–male**	**Impulsive behaviour**	20.4(6.2)	20.3(5.3)	21.4(6.8)	0.13	0.875
**BIS-female**	**Impatience**	16.5(3.4)	17.9(3.9)	18.6(3.8)	1.45	0.268
**BIS-male**	**Impatience**	16.8(4.2)	16.6(3.2)	16.8(4.3)	0.08	0.992
**AGQ- female**	**Aggression-physical**	**15.2(5.1)**	**20.1(7.4)**	**24.4(8.8)**	**6.79**	**0.002a**
**AGQ- male**	**Aggression- physical**	27.7(9.8)	23.6(6.7)	32.0(16.5)	1.87	0.167
**AGQ- female**	**Aggression-verbal**	12.0(3.8)	13.2(5.6)	14.1(7.6)	0.62	0.542
**AGQ- male**	**Aggression verbal**	14.4(5.6)	14.4(3.7)	17.8(8.0)	1.60	0.216
**AGQ-female**	**Aggression- anger**	20.6(9.4)	21.1(10.1)	28.2(10.1)	0.07	0.065
**AGQ- male**	**Aggression-anger**	23.1(9.7)	21.6(7.9)	22.7(11.0)	0.10	0.906
**AGQ- female**	**Aggression-hostility**	**24.7(12.7)**	**31.7(11.2)**	**35.5(12.0)**	**3.53**	**0.037a**
**AGQ-male**	**Aggression-hostility**	33.9(11.2)	28.9(8.4)	32.8(14.9)	0.75	0.477

a = *p* value of group comparison with Bonferroni post hoc test

#### Predicting low lethal attempters and high lethal attempters

The comparisons of physical aggression scores in Table 2 shows that females in the high lethal suicide attempt group scored higher than the low lethal suicide attempt group and ideator group. This result formed the basis for a logistic regression analysis to test the predictability of physical aggression in low lethal suicide attempters and high lethal attempters. Suicide attempters who used suicide methods with low lethality were coded as ‘0’ and suicide attempters where the suicide methods used were of high lethality were coded ‘1’. The differences in physical aggression scores between males and females, shown in Table 2, suggests gender as a control variable together with depression, which is the most common mental illness related to suicidal behavior, as a second control variable.

In Table 3, the model comparing low lethal suicide attempters and high lethal suicide attempters found the physical aggression score (*p* < 0.032) to be a contributing factor. The Nagelkerke R square was 0.13. The OR of physical aggression shows a positive relationship to high lethal attempters.

**Table 3:** Logistic regression predicting low lethal suicide attempters vs. high lethal suicide attempters

Variables	OR	Confidence Interval	*P* Value
**Gender**	1.3	0.44–4.41	0.573
**Depression**	1.0	0.92–1.08	0.936
**Aggression-physical**	1.1	1.01–1.14	0.032

## Discussion

Physical aggression score contributed, with relatively weak effect, to the difference between predicted low lethal suicide attempt and predicted high lethal suicide attempt when including gender and depression in the model. Female patients with high lethal suicide attempt scored higher on physical aggression than suicide ideators. Impulsive behaviour was significantly higher in the female attempter groups compared to the ideator group, and hostile aggression was significantly higher in the female high lethal attempt group compared to the suicide ideator group. As predicted, the impulsive behaviour score and the physical aggression score can differentiate suicide ideators from suicide attempters. This is in accordance with other studies [14–16].

These results indicate that there are some aspects of impulsive behaviour and some aspects of aggressive behaviour that can be of importance to the suicidal process and in differentiating suicide ideators from suicide attempters. It also indicates that not all aspects of the personality concepts may be of equal importance to suicidal behaviour. Similar results were found in a large sample of students, in a study that also found only some aspects of aggression and impulsivity to differentiate suicide ideators from suicide attempters [24]. Wang et al. (2014) used similar indexes of aggression and impulsivity; however, they found self-oriented attack and cognitive impulsivity to be predictors of suicide attempt when comparing student groups that were non-suicidal, suicide ideators, and suicide attempters.

It has also been found that impulsivity predicts suicide ideation but not attempt [1]. In the current study, the results suggest that the behaviour impulsivity scores of female low lethal attempters were higher than for female ideators, and there were no differences in male scores on impulsivity between the three suicide risk groups. The fact that only some aspects of impulsivity and aggression may contribute to the difference between thinking about suicide and making a lethal suicide attempt could explain why some studies find a relation [26] and some studies do not find a relation between impulsivity, aggression, and suicidal behaviour [28]. A recent and, to date, the largest meta-analysis of aggression, impulsivity, and suicidality (different measures of suicidal behaviour excluding self-harm without suicide intent) confirmed a positive relationship between impulsivity and suicidality, especially behaviour impulsivity [41]. A study of impulsivity facets in recent suicide attempters found deficits in conscientiousness to be a robust predictor of suicide likelihood [42]. A review of the link between impulsivity and suicidal behaviour found that researchers have been unable to adequately measure impulsivity of suicide attempts and that measures sensitive to episodic planning must be developed [43].

In relation to suicide capability in the 3ST, it makes sense that impulsive behaviour could play a role in the transmission from suicide thought to suicide attempt, and that higher levels of physical aggression could play a role in becoming capable of high lethal suicide attempt. If you have a tendency to act before you think of the consequences, then acting on your suicidal thoughts could be a pattern of behaviour. If you are used to expressing your aggressive feelings and thoughts physically – for example by acting out, harming others, or breaking/destroying materials – then this habituation of behaviour could make you more able to act on your suicidal ideation in a more physical or violent manner, which could be lethal. Suicide capability can be built by dispositional, acquired, and practical contributors. Research has found correlations between childhood abuse, level of aggression, and possibility of suicide [44]. It has been found that close to 50% of dual-self harmers (self-harm with suicidal intent and serious aggressive behaviours towards others), have experienced one or more adverse childhood experience [45].

The gender differences found in this study could relate to the gender paradox often referred to in the literature. The fact that men scored higher on physical aggression than women, and that higher physical aggression scores were related to high lethal attempts may be related to the paradox that men die in suicide more often than women even though women attempt suicide more often. This gender difference can also relate to suicide capability, as physical aggression can be classified as habituation to pain-induced behaviour. Research also directly indicates that men have higher levels of acquired capability for suicide compared to women [46]. Another aspect of the gender paradox and building blocks of suicide capability is that men often choose more lethal suicide methods. This preference could be a practical contributor to capability and the choice could also be related to their dispositional higher level of physical aggression.

The difference in group comparison of aggression and impulsivity was only significant for females, but gender was not found as a significant predictor in the model predicting physical aggression and depression. This combination of findings was surprising. It is possible that higher levels of physical aggression are related to higher lethality of suicide attempts for both males and females. In the analysis without the participants who had died of suicide, a significant difference on physical aggression scores was found between men in the high lethal suicide attempt group and men in the low lethal suicide attempt group; the high lethal attempters had higher levels of physical aggression.

A recent study of aggression, impulsivity, mental pain, and communication difficulties in medically serious and medically non-serious suicide attempters [26] hypothesised that aggression and impulsivity would have a catalyzing effect on the lethality of suicide attempts over and above communication difficulties and mental pain, but they found only the combination of unbearable mental pain and difficulties in communication to have a magnifying effect on the risk of lethal suicide attempt. The model of the current study, predicting physical aggression, only explains 13% of the variance. It could be that unbearable mental pain and difficulties in communication are better predictors of high lethal suicide attempt. Further investigation into different aspects of aggression in relation to lethality in suicide attempters is needed.

A limitation of this study is the relatively small sample size. It is difficult to include large samples of suicide ideators, low lethal suicide attempters, and high lethal suicide attempters because most people do not attempt suicide. The timeframe for follow-up registration of the participants who have died in suicide varied, as inclusion date varied across the sample. Furthermore, measurement of impulsivity and aggression are, in this study, based on self-report only, and it is not certain that the individuals are aware of their own impulsivity behaviour patterns or patterns of aggression. It is also difficult to make clear-cut categories of suicide ideators, low lethal suicide attempters, and high lethal suicide attempters. Suicidal behaviour can be an expression that serves different functions for individuals. For some people, suicidal behaviour can be a way to communicate pain and regulate negative emotions, while for other people it can be a way to try to escape the unbearable situation they are in, and for yet other people it can be a conscious death wish after living a difficult or meaningless life for too long. Future studies should include larger samples, look at gender differences, include several nuances of suicidal attempt, and test different aspects of aggression and impulsivity.

## Conclusion

Clinicians assessing suicidal individuals should be aware that some aspects of impulsivity, like impulsive behaviour, can be more prominent in female low lethal suicide attempters. Clinicians should address aspects of aggression like hostility, as it can be more prominent in female high lethal attempts. Gender differences in suicidal behaviour, impulsivity, and aggression could suggest that treatment of suicidal patients could be tailored differently for men and women. Suicide prevention should address physical aggression, based on the knowledge that higher levels can be associated with high lethal suicide attempt.

## Data Availability

The dataset of the current study is available from the corresponding author on reasonable request.

## Abbreviations

C-SSRS: The Columbia-suicide severity rating scale
MADRS: The Montgomery-Åsberg depression rating scale
BIS-11: Barratt impulsiveness scale
AQ: Buss & Perry aggression questionnaire
3ST: The three step theory
